# US Adults Practicing Healthy Lifestyles Before and During COVID-19: Comparative Analysis of National Surveys

**DOI:** 10.2196/45697

**Published:** 2023-03-31

**Authors:** Hon Lon Tam, Sek Ying Chair, Isaac Sze Him Leung, Leona Yuen Ling Leung, Alex Siu Wing Chan

**Affiliations:** 1 The Nethersole School of Nursing The Chinese University of Hong Kong Shatin, NT China (Hong Kong); 2 Department of Statistics The Chinese University of Hong Kong Shatin, NT China (Hong Kong); 3 The Ronin Institute Montclair, NJ United States; 4 Canadian Academy of Independent Scholars Vancouver, BC Canada; 5 Department of Applied Social Sciences The Hong Kong Polytechnic University Hung Hom, Kowloon China (Hong Kong)

**Keywords:** healthy lifestyle, health risk behaviors, habits, noncommunicable diseases, population surveillance, Behavioural Risk Factor Surveillance System, BRFSS

## Abstract

**Background:**

Practicing healthy lifestyles can reduce the risk to develop noncommunicable diseases and the related mortality. Studies showed that practicing healthy lifestyles could enhance disease-free life expectancy and preserve bodily functions. However, engagement in healthy lifestyle behavior was suboptimal.

**Objective:**

This study aimed to define individuals’ lifestyle characteristics before and during COVID-19 and determine the factors associated with practicing a healthy lifestyle. This cross-sectional study was conducted using data from the 2019 and 2021 Behavioral Risk Factor Surveillance System surveys.

**Methods:**

US individuals aged ≥18 years were interviewed via phone call. Healthy lifestyles were assessed through corresponding questions regarding the maintenance of optimal body weight, physical activity, daily consumption of at least five portions of fruits and vegetables, current smoking status, and alcohol consumption. Missing data were imputed using a package in the R statistical software. The effects of practicing a healthy lifestyle on cases without missing data and those with imputation were reported.

**Results:**

There were 550,607 respondents (272,543 and 278,064 from 2019 and 2021, respectively) included in this analysis. The rates of practicing a healthy lifestyle were 4% (10,955/272,543) and 3.6% (10,139/278,064) in 2019 and 2021, respectively. Although 36.6% (160,629/438,693) of all 2021 respondents had missing data, the results of the logistic regression analysis for cases without missing data and those with imputation were similar. Of the cases with imputation, women (odds ratio [OR] 1.87) residing in urban areas (OR 1.24) with high education levels (OR 1.73) and good or better health status (OR 1.59) were more likely to practice healthier lifestyles than young individuals (OR 0.51-0.67) with a low household income (OR 0.74-0.78) and chronic health conditions (OR 0.48-0.74).

**Conclusions:**

A healthy lifestyle should be strongly promoted at the community level. In particular, factors associated with a low rate of practice of healthy lifestyles should be targeted.

## Introduction

Noncommunicable diseases (NCDs) are chronic health conditions including cardiovascular diseases and diabetes, which were responsible for approximately 74% deaths worldwide in 2019 [1]. Practicing a healthy lifestyle reduces the risk of NCDs and associated all-cause mortality [2-5]; it has been shown to reduce 78% and 42% of incident diabetes and hypertension, respectively [2,3]. The following 5 components determine a healthy lifestyle: optimal body weight, regular physical activity, healthy diet, smoking cessation, and limited alcohol consumption. Loef and Walach [4] conducted a meta-analysis of 15 studies and reported that strict adherence to most of these components could significantly reduce all-cause mortality (ie, from 26% to 68%). These findings were supported by the results of a cohort study involving 487,198 healthy adults; after a median follow-up of 10.2 years, Zhu et al [5] reported that practicing a healthy lifestyle could reduce up to 68% of the all-cause mortality and mortality rate associated with ischemic heart disease (58%), stroke (79%), and respiratory diseases (81%). A large-scale cohort study involving 111,562 participants with 28-34 years of follow-up showed that a healthy lifestyle could enhance disease-free life expectancy at 50 years of age by up to 31.1 years [6]. Another 3-year follow-up cohort study involving 3107 older adults found that practicing a healthy lifestyle could alleviate the deterioration of gait speed and cognitive function [7]. In brief, practicing a healthy lifestyle can reduce all-cause mortality, slow down the progression of NCDs, and preserve bodily functions. Accordingly, the World Health Organization (WHO) has recommended practicing healthy lifestyles to prevent the risk of NCDs across all age groups [8].

Despite this, engagement in healthy lifestyle behaviors has remained suboptimal, with some factors hindering individuals from pursing such lifestyles. Using a random sampling technique, Azizan et al [9] investigated adherence to a healthy diet among low-income individuals in Malaysia; only 34.8% of the 1450 participants followed a healthy diet. A low level of education and diabetes prevented individuals from following a healthy diet [9]. However, a cross-sectional study conducted in Iran comprising 500 patients with diabetes revealed that over 80% of the participants followed healthy diets and were physically active; the use of convenience sampling technique might be the reason of the high adherence rate of healthy diets and physical activity [10]. Another cross-sectional study conducted in Jordan, using random sampling, involving 1000 patients with hypertension reported that only 23% of the included participants practiced healthy lifestyles; being men and having a low level of hypertension knowledge prevented the participants from practicing healthy lifestyles [11]. On the other hand, fewer rural residents practice healthy lifestyles [12,13]. This might be why a higher mortality rate was noted in the rural areas [14]. Different sociodemographic conditions, comorbidities, and residential areas may influence individuals’ decisions to practice healthy lifestyles.

A healthy lifestyle should be promoted at all ages. Studies have shown that practicing a healthy lifestyle may not always result in satisfactory outcomes and that many factors can influence individuals’ decisions to pursue such lifestyles. However, COVID-19 has markedly altered our living environments and behaviors [15-17]. For example, a 3-month longitudinal study found that people spent less time visiting public places and became physical inactivity during COVID-19 [18]. Previous studies assessing healthy lifestyles were conducted at the community level and before the pandemic was announced. Our main objectives were to (1) define the individuals’ characteristics and their practice of healthy lifestyles in 2019 and 2021 and (2) determine the factors associated with practicing a healthy lifestyle.

## Methods

This cross-sectional study was reported in accordance with the Strengthening the Reporting of Observational Studies in Epidemiology guideline [19]. The study was performed using publicly available data from the Centers for Disease Control and Prevention (CDC).

### Data Sources

Secondary analysis of data from the 2019 and 2021 Behavioral Risk Factor Surveillance System (BRFSS) surveys, which are national annual surveys that investigate health-related behaviors and health conditions of noninstitutionalized US adults aged ≥18 years, was performed [20]. The surveyor used the random-digit–dialing technique to generate random phone numbers for telephone and mobile phone interviews across 50 states in the United States, the District of Columbia, Guam, Puerto Rico, and the US Virgin Islands. The latest BRFSS survey was conducted in 2021. The median survey response rate of the 2019 BRFSS survey and 2021 BRFSS survey was 49.4% and 44%, respectively [20,21].

### Study Population

Cases for which data regarding sociodemographic variables or chronic health conditions were missing and individuals who refused to answer questions were excluded from the study. We included 272,543/418,268 (65.2%) and 278,064/438,693 (63.4%) individuals from the 2019 and 2021 BRFSS 2021 surveys, respectively.

### Variables

#### Sociodemographic Characteristics and Perceived Health

Following previous studies [9-12], several sociodemographic variables and the perceived health were extracted from the BRFSS surveys. Marital status, employment status, and housing were recategorized in this study, whereas the rest followed the categorizations from the BRFSS survey. In addition, some variables were recategorized in the logistic regression analysis, where perceived health was categorized as “fair or poor” (including the rating of fair and poor) and “good or better” (including the rating of good, very good, and excellent) and education level was recategorized as “high school or less” (including attended and graduated from high school) and “college and above” (including attended and graduated from college).

#### Healthy Lifestyle

A healthy lifestyle was defined by adherence to healthy behaviors and the avoidance of risky behaviors. Based on the items in the BRFSS and WHO recommendations [8], health behaviors were determined by three components: (1) optimal body weight, defined by a no response to the item “adults who have a body mass index greater than 25”; (2) physical activity, defined by the item “adults who reported doing physical activity or exercise during the past 30 days other than their regular job”; and (3) healthy diet, defined by the reports of the daily consumption of at least five portions of fruits and vegetables.

Following WHO recommendations [8], two risky behaviors were investigated: (1) current smoker status, which was directly surveyed using the BRFSS; and (2) excessive alcohol users, defined as heavy or binge drinkers.

#### Chronic Health Conditions

Several chronic health conditions, namely hypertension, high cholesterol, diabetes, asthma, chronic obstructive pulmonary disease, and depression, were assessed using the BRFSS survey data.

### Statistical Analysis

Data were analyzed using the R statistical software (R Foundation for Statistical Computing). Logistic regression analysis was used to investigate which independent variables were associated with practicing healthy lifestyles (dependent variable). These independent variables included the sociodemographic characteristics, health status, and health-related behaviors. To inform future research, the latest 2021 data set was analyzed. Because the percentage of the excluded subjects was as high as 36.6% and the missing rate of outcome variables were associated with various covariates (missing at random instead of missing at completely random), multiple imputations were conducted to estimate the missing variables among the excluded subjects. Five imputed data sets were created by the *multiple imputation via chained equations (MICE)* package in R.

MICE imputation starts by a simple imputation such as mean imputations on all variables with missing value. Then, these imputed missing values are updated by running a series of regressions, whereby each variable with missing value is regressed upon other variables repeatedly until convergence. For cases with imputations, logistic regression analyses were performed for each of the imputed data sets. The regression coefficients were combined using the *pool()* function within the *MICE* package. In addition, we used the *detectseparation* package in R to check if there was any multicollinearity in our logistic regression analysis. The results of the 2021 data set logistic regression analyses were presented both for cases without missing data and for those with imputations. A *P* value <.05 was considered statistically significant.

### Ethical Considerations

The surveys were conducted by US federal agencies where verbal consent was obtained from each participant prior to the data collection [20,21]. The BRFSS data set are allowed for secondary data analysis without permission required. No institutional review board approval or patient informed consent was required for secondary data analysis. The participants’ personal information had been deidentified in the data set prior to being publicly available [20,21].

## Results

### Individual Characteristics

We analyzed 550,607 respondents (272,543 and 278,064 from the 2019 and 2021 BRFSS surveys, respectively; Table 1). The majority of the respondents were female (n=284,876, 51.7%) and resided in urban areas (n=468,547, 85.1%); 35.2% (n=193,850) of the respondents were aged 65 years and older. Regarding health and related behaviors, 83% (n=456,846) of individuals were perceived to have a good or better health status. Hypertension (n=276,148, 50.2%) and high cholesterol (n=248,440, 45.1%) were the most commonly reported chronic diseases. The percentage of respondents who performed physical activity was greater in 2021 than in 2019 (214,943/278,064, 77.3% vs 204,733/272,543, 75.1%); in contrast, the percentage of smokers was lower in 2021 than in 2019 (36,983/278,064, 13.3% vs 38,662/272,543, 14.2%). The percentage of practicing a healthy lifestyle nonsignificantly decreased from 4% in 2019 to 3.6% in 2021.

**Table 1:** Individual characteristics among complete cases without missing data in 2019 and 2021.

Variables				2019 (n=272,543), n (%)		2021 (n=278,064), n (%)
**Sociodemographic**						
	**Sex**					
		Male	129,986 (47.7)		135,745 (48.8)	
		Female	142,557 (52.3)		142,319 (51.2)	
	**Age range (years)**					
		18-24	13,910 (5.1)		11,020 (4)	
		25-34	28,659 (10.5)		28,729 (10.3)	
		35-44	34,174 (12.5)		39,392 (14.2)	
		45-54	41,268 (15.1)		44,794 (16.1)	
		55-64	58,183(21.4)		56,628 (20.3)	
		65 and older	96,349 (35.4)		97,501 (35.1)	
	**Residential area**					
		Urban	230,874 (84.7)		237,673 (85.5)	
		Rural	41,669 (15.3)		40,391 (14.5)	
	**Education level**					
		Attended high school or less	14,805 (5.4)		13,065 (4.7)	
		Graduated from high school	67,247 (24.7)		65,609 (23.6)	
		Attended college or above	77,622 (28.5)		77,630 (27.9)	
		Graduated from college or above	112,869 (41.4)		121,760 (43.8)	
	**Marital status**					
		Single	52,796 (19.4)		54,306 (19.5)	
		Married	146,251 (53.7)		152,435 (54.8)	
		Divorced, widowed, or others	73,496 (27)		71,323 (25.6)	
	**Employment**					
		Employed	145,075 (53.3)		152,050 (54.7)	
		Unemployed	27,083 (9.9)		26,550 (9.5)	
		Retired or unable to work	100,385 (36.8)		99,464 (35.8)	
	**Household income (US $)**					
		Less than 15,000	22,051 (8.1)		15,772 (5.6)	
		15,000 to <25,000	39,395 (14.5)		26,772 (9.6)	
		25,000 to <35,000	26,803 (9.8)		33,851 (12.2)	
		35,000 to <50,000	37,991 (13.9)		38,514 (13.9)	
		50,000 or more	146,303 (53.7)		163,155 (58.7)	
	**Housing**					
		Own	199,188 (73.1)		206,029 (74.1)	
		Rent	62,790 (23)		62,854 (22.6)	
		Other arrangement	10,565 (3.9)		9181 (3.3)	
	**Health insurance**					
		Yes	252,202 (92.5)		264,984 (95.3)	
		No	20,341 (7.5)		13,080 (4.7)	
**Health and related behaviors**						
	**Perceived health**					
		Excellent	44,293 (16.3)		49,072 (17.6)	
		Very good	94,103 (34.5)		98,468 (35.4)	
		Good	84,798 (31.1)		86,112 (31)	
		Fair	36,038 (13.2)		33,517 (12.1)	
		Poor	13,311 (4.9)		10,895 (3.9)	
	**Chronic health condition**					
		Hypertension	110,699 (40.6)		165,449 (59.5)	
		High cholesterol	95,634 (35.1)		152,806 (55)	
		Diabetes	37,048 (13.6)		37,910 (13.6)	
		Asthma	25,755 (9.4)		27,300 (9.8)	
		COPD^a^	22,545 (8.3)		22,261 (8)	
		Depression	53,900 (19.8)		57,284 (20.6)	
	**Report of comorbidities**					
		None	91,235 (33.5)		29,563 (10.6)	
		2 and more	101,630 (37.3)		159,570 (57.4)	
	**Health behaviors**					
		Maintaining optimal body weight	83,875 (30.8)		80,754 (29)	
		Performing physical activity	204,733 (75.1)		214,943 (77.3)	
		Healthy diet	44,028 (16.2)		40,444 (14.5)	
	**Risk behaviors**					
		Current smoker	38,662 (14.2)		36,983 (13.3)	
		Excessive alcohol user	44,171 (16.2)		44,798 (16.1)	
	**Practicing healthy lifestyles**					
		Yes	10,955 (4)		10,139 (3.6)	
		No	261,590 (96)		267,925 (96.4)	

^a^COPD: chronic obstructive pulmonary disease.

### Predictors of Practicing Healthy Lifestyles

Table 2 shows the results of the logistic regression analysis for cases without missing data in 2019 and 2021 who practiced healthy lifestyles; similar results were obtained for 2019 and 2021. In 2021, female individuals (odds ratio [OR] 1.95; *P*<.001) who had a high level of education (OR 1.99; *P*<.001), were single (OR 1.10; *P*=.02), unemployed (OR 1.10; *P*=.001), and had perceived good health (OR 1.62; *P*<.001) were found to have a higher likelihood of practicing healthy lifestyles. Compared with adults aged 65 years and above, young adults aged 18-64 years tended not to practice healthy lifestyles (OR 0.53-0.66; *P*<.001). Individuals with a low household income have a low likelihood of practicing healthy lifestyles (OR 0.72-0.79; *P*<.001).

Individuals with chronic health conditions had a low likelihood of practicing healthy lifestyles. The data in Table 2 show that individuals with NCDs such as hypertension and diabetes had a low likelihood of practicing healthy lifestyles (OR 0.48-0.83; *P*<.001).

For the cases with imputations, the weighted estimates of the logistic regression analysis results from 5 imputed data sets are presented in Table 3 (n=438,693). The rates of missing outcomes of healthy lifestyles in each variable are reported in Table S1 in Multimedia Appendix 1; briefly, the rate varied from 14.1% (61,858/438,693) to 27.3% (119,823/438,693). The weighted estimates were in line with those for cases without missing data (Table 2). Multicollinearity was not detected in all regression analyses.

**Table 2:** Logistic regression analysis of practicing a healthy lifestyle among complete cases without missing data in 2019 and 2021.

Variables		2019 (n=272,543)			2021 (n=278,064)	
		Odds ratio	*P* value	Odds ratio		*P* value
Sex: female		2.03	<.001	1.95		<.001
Residence in urban areas		1.22	<.001	1.23		<.001
Education: college and above		1.90	<.001	1.99		<.001
**Age range (years)**						
	18-24	0.80	<.001	0.59		<.001
	25-34	0.55	<.001	0.53		<.001
	35-44	0.54	<.001	0.58		<.001
	45-54	0.56	<.001	0.54		<.001
	55-64	0.69	<.001	0.66		<.001
	65 and above	Reference		Reference		
**Marital status**						
	Single	0.92	.09	1.10		.02
	Married	1.06	.03	1.15		<.001
	Divorced, widowed, or others	Reference		Reference		
**Employment**						
	Employed	0.87	<.001	0.86		<.001
	Unemployed	1.17	<.001	1.11		.001
	Retired or unable to work	Reference		Reference		
**Household income (US $)**						
	Less than 15,000	0.66	<.001	0.78		<.001
	15,000 to <25,000	0.72	<.001	0.72		<.001
	25,000 to <35,000	0.71	<.001	0.77		<.001
	35,000 to <50,000	0.72	<.001	0.79		<.001
	50,000 or more	Reference		Reference		<.001
Perceived good or better health		1.51	<.001	1.62		<.001
Hypertension		0.51	<.001	0.49		<.001
High cholesterol		0.74	<.001	0.71		<.001
Diabetes		0.49	<.001	0.48		<.001
Asthma		0.84	<.001	0.83		<.001
COPD^a^		0.68	<.001	0.58		<.001
Depression		0.73	<.001	0.71		<.001

^a^COPD: chronic obstructive pulmonary disease.

**Table 3:** Logistic regression analysis of practicing a healthy lifestyle among all cases with multiple imputation in 2021 (n=438,693).

Variables		Β (SE)	Wald chi-square (*df*)	Odds ratio (95% CI)
Sex: female		0.628 (0.019)	1064.00 (1)	1.87 (1.80-1.95)^a^
Residence in urban areas		0.212 (0.026)	66.32 (1)	1.24 (1.17-1.30)^a^
Education: college and above		0.551 (0.025)	478.90 (1)	1.73 (1.65-1.82)^a^
**Age range (years)**				
	18-24	–0.401 (0.047)	72.59 (1)	0.67 (0.61-0.73)^a^
	25-34	–0.667 (0.037)	321.8 (1)	0.51 (0.47-0.55)^a^
	35-44	–0.578 (0.036)	258.80 (1)	0.56 (0.52-0.60)^a^
	45-54	–0.640 (0.037)	295.50 (1)	0.53 (0.49-0.46)^a^
	55-64	–0.453 (0.029)	246.20 (1)	0.64 (0.60-0.67)^a^
	65 and above	Reference		
**Marital status**				
	Single	0.029 (0.030)	0.91 (1)	1.03 (0.97-1.09)
	Married	0.098 (0.025)	15.55 (1)	1.10 (1.05-1.16)^a^
	Divorced, widowed, or others	Reference		
**Employment**				
	Employed	–0.148 (0.027)	30.58 (1)	0.86 (0.81-0.90)^a^
	Unemployed	0.114 (0.033)	11.64 (1)	1.12 (1.05-1.20)^a^
	Retired or unable to work	Reference		
**Household income (US $)**				
	Less than 15,000	–0.294 (0.082)	12.70 (1)	0.75 (0.63-0.87)^b^
	15,000 to <25,000	–0.302 (0.099)	9.35 (1)	0.74 (0.61-0.89)^c^
	25,000 to <35,000	–0.267 (0.042)	39.45 (1)	0.77 (0.70-0.83)^a^
	35,000 to <50,000	–0.245 (0.034)	52.58 (1)	0.78 (0.73-0.83)^a^
	50,000 or more	Reference		
Perceived good or better health		0.465 (0.034)	191.60 (1)	1.59 (1.49-1.70)^a^
Hypertension		–0.674 (0.022)	917.20 (1)	0.51 (0.48-0.53)^a^
High cholesterol		–0.307 (0.019)	254.90 (1)	0.74 (0.70-0.76)^a^
Diabetes		–0.729 (0.04)	339.60 (1)	0.48 (0.44-0.52)^a^
Asthma		–0.168 (0.034)	23.92 (1)	0.85 (0.79-0.90)^a^
COPD^d^		–0.500 (0.048)	106.90 (1)	0.61 (0.55-0.66)^a^
Depression		–0.347 (0.025)	197.60 (1)	0.71 (0.67-0.74)^a^

^a^Significant result: *P* value <.001

^b^Significant result: .001≤ *P* value <.01

^c^Significant result: .01≤ *P* value <.05

^d^COPD: chronic obstructive pulmonary disease.

## Discussion

### Principal Findings

This study assessed the determinants of practicing healthy lifestyles at the national level using 2019 and 2021 BRFSS survey data. The distribution of sociodemographic variables and health and related behaviors were similar for both the surveys. However, the tendency to practice healthy lifestyles slightly decreased in 2021. The logistic regression analysis results indicated that female individuals residing in urban areas with a higher level of education were more likely to practice healthy lifestyles, whereas younger individuals with a low household income were less likely to do so. Although chronic health conditions hindered individuals from practicing healthy lifestyles, those with perceived good or better health were more likely to practice healthy lifestyles.

However, the overall rate of practicing a healthy lifestyle was still very low. Fang et al [22] analyzed 2013 BRFSS data and reported that only 1.7% of the assessed individuals with hypertension practiced healthy lifestyles. Other studies have shown that 2.1% of healthy adults and 6% of health care providers practiced healthy lifestyles [5,6]. Moreover, although more individuals performed physical activity, the percentage of individuals with optimal body weights and following healthy diets decreased. High BMI and unhealthy diet were major risk factors for disability and premature death worldwide [23,24]. In this study, we found that the individuals’ residences considerably affected their decisions to practice healthy lifestyles. Rural residents reported that they received limited access to health-related information from health care providers [25]. Although the overall number of health care providers, such as physicians, nurse practitioners, and physician assistants, in the United States significantly increased between 2009 and 2017, the urban-rural disparities remained significant; the number and density of health care providers increased to a greater extent in urban areas than in rural areas [26]. Therefore, a healthy lifestyle should be strongly promoted, and urban-rural health disparities should be tackled urgently.

In concordance with previous studies [9,11,27], our results showed that individuals with a low household income, who were male, and had high school or lower levels of education were less likely to practice healthy lifestyles. Although married people were more likely to practice healthy lifestyles, results from another national survey revealed that such individuals were more likely to be overweight or obese [28]. In contrast, we found that young people (aged <65 years) were less likely to practice healthy lifestyles. Thus, early health promotion is required because the risk of developing NCDs exponentially increases after 50 years of age [29].

Practicing healthy lifestyles among individuals with hypertension were focused because hypertension has been the leading risk factor for global disease burden worldwide for a decade [23,30]. Although certain guidelines recommend healthy lifestyles and medical treatments to effectively control blood pressure [31-33], our analysis showed that individuals with hypertension were less likely to practice healthy lifestyles. A Finnish cohort study involving 41,225 participants followed up over 4 years reported that the initiation of antihypertensive treatment significantly decreased the tendency of individuals with hypertension to practice healthy lifestyles (OR 0.55-0.92) [34]. As blood pressure can indeed be controlled with medications, less attention has been paid to individuals with hypertension practicing healthy lifestyles. Approximately 82.3% of individuals with hypertension in the United States use antihypertensive medications [35]; they might not practice healthy lifestyles as their blood pressure is medically controlled.

Concern about around those with diabetes practicing healthy lifestyles have been raised, because these individuals showed the least likelihood of doing so. A healthy lifestyle has been indicated in various diabetes management guidelines [36,37]. However, our findings revealed that individuals with diabetes were the least likely to practice healthy lifestyle, showing the smallest OR among all variables. Individuals with diabetes have emphasized that education and support from health care providers can enhance their self-care tendencies [38]. Hence, new methods for diabetes care should be used.

### Implications

Our study findings have strong implications for clinical practice, research, and policy making. The low likelihood of practicing healthy lifestyles highlights the need for strengthening current practices. Health education supported by printed materials, phone calls, and SMS text messaging may improve individuals’ adherence to healthy lifestyles and medical treatments [39-43]. Given the rapid increase in smartphone ownership and advancing technologies [44], digital interventions such as website and smartphone apps have been shown to slightly improve adherence to a healthy lifestyle [45-47]. Supportive methods and digital interventions can be integrated into current health promotion activities to facilitate healthy lifestyles and widen the possibility of receiving health-related information and support from health care providers, especially for rural residents. Moreover, digital interventions can help overcome the barriers to public health measures caused by COVID-19 [18]. However, we noted that several sociodemographic characteristics can hinder individuals from practicing healthy lifestyles. Additionally, there may be associations between depression, ethnic background, and socioeconomic status [48]. Future studies can explore the associations and focus on developing specific interventions that promote healthy lifestyles among such individuals.

Rural residents showed a low likelihood of practicing healthy lifestyles; this highlights the need of support from policy makers. A cohort study of 25,014 middle-age and older adults with a 20-year follow-up revealed that performing any type of physical activity could significantly reduce the likelihood of hospitalization to a mean of 0.42 days per year; this reduced likelihood of hospitalization could reduce health care expenditures by approximately 7% [49]. Moreover, some amount of the saved health care expenditure could be granted as tax deductions to individuals who participate in healthy lifestyle programs and provide evidence of practicing such lifestyles (ie, reduced body weight). Such tax deductions can attract more people toward healthy lifestyles, further decreasing the overall health care expenditure. The shortage of health care providers may be managed through the use of digital interventions for providing education and support to residents in rural areas. However, more interventions should be developed to attract health care providers to work and remain in rural areas. For example, increased training in rural areas, the payment of locum relief, and loan repayment may help do so [50].

### Limitations

This study has 4 key limitations. First, the variables in the secondary analysis were limited to the data set used. For example, only 1 question in the data set investigated the individuals’ physical activity habits. Moreover, the question was rather general and inquired whether the individuals performed any type of physical activity or exercise during the past 30 days. Two essential components—frequency and intensity—were not explored. Thus, our study findings might not adequately represent the level of physical activity as recommended by the WHO [51]. Second, all variables were reported by individuals via phone call. Therefore, we might have encountered reporting biases arising from the participant’s recall from memory, and the interviewers may have recorded the responses incorrectly. To address such issues, the CDC has created a program to identify errors or conditions of concern due to data entry by the interviewers [20,21]. Third, although the phone numbers used in each year were not repeated, it was unclear if some numbers would be used in the other survey years. Hence, it was possible that some people might have responded to both surveys in this study. Fourth, the overall rate of missing data was high at 36.6% in 2021, which could have impacted the generalizability of the results. However, we performed multiple imputations, and the pooled results of the imputed data were in line with those from the cases without missing outcomes. Hence, our imputed data provided an accurate estimation of the influence of sociodemographic variables and chronic health conditions on practicing healthy lifestyles and enhanced the generalizability of our findings.

### Conclusions

Practicing a healthy lifestyle can have numerous health benefits across all age groups. We found that very few people practiced healthy lifestyles before and during COVID-19. Some sociodemographic variables and chronic health conditions were identified as significant factors affecting individuals’ decisions to practice healthy lifestyles. Several interventions were suggested to strengthen the promotion of healthy lifestyles at the community level and to attract more health care providers to work and remain in rural areas.

## Appendix

Multimedia Appendix 1 Missing outcome of healthy lifestyles in each variable.

## Abbreviations

BRFSS: Behavioral Risk Factor Surveillance System
CDC: Centers for Disease Control and Prevention
MICE: multiple imputation via chained equations
NCD: noncommunicable disease
OR: odds ratio
WHO: World Health Organization

## Data Availability

The data used in this study (Behavioral Risk Factor Surveillance System [BRFSS] 2019 and 2021) are publicly available [20,21].
