# Supplementary material for: US Adults Practicing Healthy Lifestyles Before and During COVID-19: Comparative Analysis of National Surveys
Source: JMIR Public Health Surveill. 2023 Mar 31;9:e45697. doi: 10.2196/45697 (PMC10131672; doi:10.2196/45697)
Supplement: Multimedia Appendix 1 [file publichealth_v9i1e45697_app1.docx]

**Table S1. Missing outcome of healthy lifestyles in each variable**

| **Variables** | **Missing rate** |
| --- | --- |
| Sex  Male  Female | 20.7%  24.9% |
| Age  18-24  25-34  35-44  45-54  55-64  65 and older | 23.1%  24.0%  23.2%  24.5%  20.8%  23.0% |
| Residential area  Urban  Rural | 23.3%  21.9% |
| Education level  High school or less  College and above | 20.5%  27.3% |
| Marital status  Single  Married  Divorced/Widowed/Others | 23.6%  21.5%  23.6% |
| Employment  Working  Not working  Retired or unable to work | 21.0%  24.0%  22.5% |
| Household income (USD)  Less than $15,000  $15,000 to < $25,000  $25,000 to < $35,000  $35,000 to < $50,000  $50,000 or more | 22.5%  21.3%  20.0%  17.7%  14.1% |
| Perceived health  Good or better health  Fair or poor health | 22.5%  24.9% |
| Chronic health condition  Hypertension  High cholesterol  Diabetes  Asthma  COPD  Depression | 22.5%  20.5%  22.6%  23.0%  23.0%  19.9% |

COPD: chronic obstructive pulmonary disease.
